# Purine Nucleotides in the Regulation of Brown Adipose Tissue Activity

**DOI:** 10.3389/fendo.2020.00118

**Published:** 2020-03-10

**Authors:** Andrea Bast-Habersbrunner, Tobias Fromme

**Affiliations:** ^1^Chair of Molecular Nutritional Medicine, TUM School of Life Sciences, Technical University of Munich, Munich, Germany; ^2^EKFZ - Else Kröner-Fresenius Center for Nutritional Medicine, Technical University of Munich, Munich, Germany

**Keywords:** brown adipose tissue, uncoupling protein 1, nucleotides, non-shivering thermogenesis, mitochondria, GMP reductase, AMP deaminase

## Abstract

Non-shivering thermogenesis in mammalian brown adipose tissue is a powerful mechanism to defend normothermia in cold climates. To minimize the loss of chemical energy, the central functional component, mitochondrial uncoupling protein 1, UCP1, must be tightly regulated. The canonical pathway of UCP1 activation includes lipolytic release of free fatty acids in response to an adrenergic signal. Activating fatty acids overcome constitutive inhibition of UCP1 by the di- and triphosphate forms of purine nucleotides, i.e., ATP, ADP, GTP, and GDP. Cellular concentrations of inhibitory, free nucleotides are subject to significant, adrenergically induced alterations. The regulatory components involved may constitute novel drug targets to manipulate brown fat thermogenesis and thereby organismic energy balance. We here review evidence for and against a dominant role of nucleotides in thermogenic control, describe conceptual routes to endogenously and pharmacologically alter free nucleotide pool size, speculate on a signaling role of degradation products released from active brown fat, and highlight gaps in our understanding of signaling and metabolic pathways involved.

## Introduction

Brown adipose tissue (BAT) provides a mechanism for adaptive, non-shivering thermogenesis to endotherm mammals [reviewed in (1)]. Isolated mitochondria from this tissue display intense oxygen consumption in the absence of ATP production, i.e., uncoupled respiration. The di- and triphosphate forms of purine nucleotides (ATP, ADP, GTP, and GDP) have long been known to restore respiratory control to such mitochondrial preparations (2–4). The nucleotide binding site was found to reside within the central thermogenic protein of the mitochondrial inner membrane later named uncoupling protein 1 (UCP1) (5–7). Nucleotides proved a constitutive inhibitor of UCP1 mediated proton conductance of the mitochondrial inner membrane and thus constitute the default shut-off mechanism in the absence of thermogenic demand. Upon BAT activation by the sympathetic nervous system, lipolytically liberated free fatty acids are thought to displace nucleotides from UCP1 in a competitive manner to act as thermogenic activators (8–10). Challenging this concept of mere competitive relief of UCP1 inhibition, several lines of evidence support an additional, active regulation of purine nucleotides in response to adrenergic stimulation.

## Nucleotides Are Actively Controlled Regulators of Ucp1 Mediated Thermogenesis

For decades, the divergent roles of actively controlled free fatty acid levels as UCP1 activators and passively acting nucleotides as constitutively present inhibitors remained the widely accepted model of thermogenic regulation in BAT. The arguments seemed obvious enough: millimolar cytosolic concentration of ATP alone seemed sufficient to fully and efficiently shut down UCP1 function at all times, exceeding the nanomolar or low micromolar dissociation constant by several orders of magnitude (11–13). To actively release the inhibitory effect of purine nucleotides on UCP1, their concentrations would need to be tremendously reduced, which seemed prohibitively wasteful, as resynthesis of one nucleotide would demand an energy investment equivalent to 50 ATP (14). However, considerable degradation of nucleotides also occurs routinely in contracting muscle, where ADP is degraded in order to preserve the ATP/ADP ratio in conditions of high ATP hydrolysis (15, 16). Furthermore, in active brown adipocytes, where large amounts of chemical energy are released as heat instead of being converted into ATP, energy efficiency does not appear a priority.

Remaining doubts were convincingly countered and brought to the point in a publication by Klingenberg, in which he finds that “the conclusion that nucleotides do not play a role in intracellular regulation of UCP1 because of a too high affinity, seems to be unfounded” (17). Arguments put forward were, first, the phospholipid composition of the mitochondrial inner membrane is very different from the phosphatidylcholine proteoliposomes in which UCP1 was initially characterized (11). Adding phosphatidylserine and especially cardiolipin not only changed absolute UCP1 activity but in particular attenuated the inhibitory potential of purine nucleotides in a dose dependent manner (17). At the 12% molar amount cardiolipin typically found in the inner membrane of BAT mitochondria (18, 19), the dissociation constant must be expected to be increased by more than an order of magnitude. Second and even more important, UCP1 is only inhibited by free, non-complexed nucleotides (20). The preferred form to be used as substrate by ATPases, however, is a complex with one of the divalent cations magnesium and calcium and accordingly, the vast majority of cytosolic purine nucleotides is cation complexed and irrelevant for the inhibition of UCP1 (21, 22). Furthermore, total purine nucleotide concentration is relatively low in brown adipocytes, e.g., by a factor of two to six compared to white adipocytes or skeletal muscle cells, respectively (23). Taking into account both a higher than anticipated dissociation constant and a lower than anticipated free nucleotide concentration, further modulation of nucleotide levels appears a plausible regulator of UCP1 activity.

If active regulation of free nucleotides is assumed to be implicated in the activation of UCP1 mediated non-shivering thermogenesis, brown adipocytes require a mechanism to reduce their concentration in response to an adrenergic stimulus, either by a shift in the balance between free and complexed nucleotides or by an altered overall amount. There is evidence for both.

**Figure 1:**
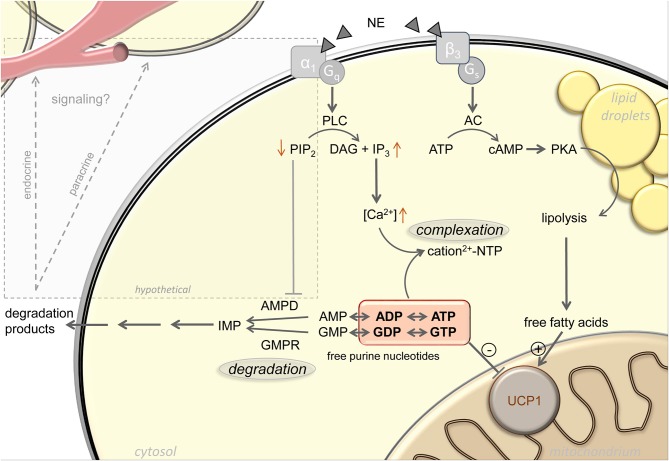
Active regulation of free purine nucleotides upon adrenergic stimulation of brown adipocytes. Norepinephrine (NE) stimulation of brown adipocytes mediates activation of uncoupling protein 1 (UCP1) via increasing free fatty acid and decreasing free purine nucleotide levels. Lipolytic release of free fatty acids occurs in response to G_s_-coupled β_3_-adrenergic receptor activation. The pool of free purine nucleotides is regulated via complexation and degradation mediated by G_q_-coupled α_1_-adrenergic receptor signaling. Phospholipase C (PLC), phosphatidylinositol 4,5-bisphosphate (PIP2), diacylglycerol (DAG), inositol 1,4,5-trisphosphate (IP3), adenylyl cyclase (AC), protein kinase A (PKA), AMP deaminase (AMPD), GMP reductase (GMPR), inosine monophosphate (IMP), adenosine mono-/di-/triphosphate (AMP/ADP/ATP), guanosine mono-/di-/triphosphate (GMP/GDP/GTP). Black arrows represent established signaling pathways, gray box and arrows indicate hypothetical pathways.

Complexation of purine nucleotides with magnesium increases with increasing cytosolic pH, which is indeed elevated upon α*-*adrenergic stimulation of brown adipocytes (21, 24–26). In addition, the inhibitory potency of free purine nucleotides on UCP1 is also a function of pH, with decreased purine affinity in response to pH elevation (20, 24). Furthermore, after thermogenic activation of brown adipocytes, total calcium content of these cells increases 15-fold by uptake from the external medium (23). Cytosolic concentrations of both calcium and magnesium sharply increase, both by uptake from the extracellular medium and release from intracellular stores (27–30). The concentration of remaining free, non-complexed purine nucleotides will strongly decrease accordingly (Figure 1).

A first indication for cold induced changes in BAT purine nucleotide concentrations themselves came from the observation of drastic changes in the transcript abundance of purine metabolism gene products. For instance, GMP reductase (Gmpr) expression is strongly and very quickly induced in BAT by cold exposure, both on the transcript and the protein level (31, 32). At peak expression after ~24 h of cold exposure, Gmpr is among the 50 most abundant transcripts in murine BAT, together with core functional components including Ucp1, citrate synthase and subunits of respiratory chain complexes [GEO accession GSE119452 (23)]. These changes in gene expression are far from incidental. Total nucleotide pool size is essentially a function of nucleotide monophosphate degradation, because mono-, di- and triphosphate forms are mutually interconvertible not only by classical ATPases, but dominantly by multiple, widely expressed adenylate- and guanylate kinases. These enzymes bi-directionally convert two diphosphates into one tri- and one monophosphate, thereby constantly rearranging the ratios between mono-, di- and triphosphate nucleotides (33). Indeed, an adrenergic stimulus leads to a loss of purine nucleotides specifically in brown adipocytes, especially ATP, ADP, and GTP. The sum of UCP1-inhibiting di- and triphosphate purine nucleotides drops to nearly one half of control levels. In accordance with gene expression data, this loss is not merely a transition toward GMP and AMP, but an actual decrease in total purine nucleotide pool size associated with a release of the respective breakdown products [(23); Figure 1].

In summary, even under control conditions, inhibitory purine nucleotide concentrations in the cytosol of brown adipocytes are in the range of their UCP1 dissociation constant. Adrenergic stimulation of non-shivering thermogenesis decreases both total pool size by enzymatic degradation and the fraction of inhibitory free nucleotides by changes in calcium concentration and pH. Activation of UCP1 includes the concerted action of both free fatty acid liberating and nucleotide degrading processes.

## Control of Nucleotide Metabolism in Brown Adipocytes

Adrenergic stimulation leads to degradation of purine nucleotides in brown adipocytes contributing to the activation of UCP1 mediated thermogenesis. Enzymes involved in purine metabolism are targets of a large number of approved and experimental drugs for indications as diverse as gout [e.g., allopurinol (34)], viral infection [e.g., ribavirin (35)], post-transplantive immunosuppression [e.g., mycophenolate (36)], and Alzheimer's disease [e.g., lumacaftor (37)]. Some of these are widely used, well-studied and feature favorable safety profiles. At least for mycophenolate, we demonstrated interference with thermogenic activation in brown adipocytes (23). Whether this phenomenon is an unexpected side effect of this and other such drugs *in vivo* remains to be studied. If so, inhibitors of human brown fat thermogenesis may be considered for the treatment of cachexia, a progressive body mass loss in cancer patients recently linked to brown fat energy wasting (38–40).

Therapeutic targeting of human brown fat thermogenesis, however, is more often discussed in the context of metabolic disease and envisions activation, not inhibition, of the immense oxidative capacity of this tissue. At least young adults feature an amount of brown adipose tissue that, fully activated, would cause a sizeable negative shift in energy balance (41), potentially exploitable to combat obesity (42), diabetes (10, 43, 44), dyslipidemia (45), and hyperphagia (46). Neither thermogenic activation, nor brown adipose tissue specific action is plausibly achieved with inhibitors of purine nucleotide metabolism. More promising targets may be found in the intracellular signaling components connecting adrenergic receptors with purine degrading enzymes, a pathway completely unresolved and of immense interest for future study.

Adrenergic stimulation leads to purine nucleotide loss in brown adipocytes. Mere changes in phosphorylation state between ADP/ATP and GDP/GTP can bi-directionally be caused by a large variety of enzymes and pathways. Similarly, the monophosphate forms AMP and GMP are readily formed or converted back to diphosphates by adenylate and guanylate kinases. Immediately upon thermogenic activation, formation of the second messengers cAMP and cGMP may, to some extent, contribute to a reduction in ATP and GTP concentration before being eliminated to AMP and GMP (47). Since cytosolic ATP/GTP and ADP/GDP together are orders of magnitude more abundant than AMP, any meaningful change in these UCP1-inhibting nucleotides by dephosphorylation alone would cause a most dramatic AMP accumulation impossible to overlook but not observed (23). Thus, total pool size of all adenine and guanine nucleotides must decrease. In order to leave the total pool of adenine and guanine nucleotides entirely, metabolites have to pass one of the two gatekeeper enzymes GMPR or AMP deaminase (AMPD), respectively (Figure 1). These two enzymes must be considered central players in Ucp1 regulation, and expression of both is indeed cold induced (31, 32).

Since full activation of UCP1 mediated thermogenesis and changes in nucleotide concentration occur rapidly upon adrenergic stimulation (23, 48, 49), the activity of gatekeepers GMPR and AMPD must be expected to be regulated on the post-translational level. To our knowledge, the regulation of mammalian GMPR activity is poorly understood, at least by anything beyond its substrate, product and highly related metabolites, in particular not by any known protein modifications or interactors (50). With its prominent role in preserving ATP/ADP ratio in contracting skeletal muscle, AMPD regulation is far better studied and understood (15, 16, 51, 52). Isoforms of AMPD are expressed from three genes, all of which well-detectable in brown adipose tissue on the transcript level [GEO accession GSE119452 (53)]. Similar to GMPR, AMPD activity is a function of nucleotide concentrations, i.e., it is activated by ADP, inhibited by ATP and therefore effectively regulated by changes in ATP/ADP ratio. Adrenergically stimulated uncoupling of respiration from ATP synthesis in brown adipocytes drastically reduces mitochondrial ATP synthesis, reducing the ATP/ADP ratio and increasing GMPR and AMPD activities, thereby enhancing nucleotide degradation (54). In striated muscle, multiple protein-protein interactions have been reported, mostly with dominant functional component of muscle contraction, e.g., myosin and troponin, some of which appear to modify kinetic properties [reviewed in (55)]. While these interactions are a plausible mechanism to couple AMPD activity to contraction, and thus rapid ATP loss, in muscle where they are exceedingly abundant, they seem unlikely to mediate adrenergically induced activity changes in brown fat.

More promisingly, facultative binding of AMPD to the plasma membrane strongly impedes catalytic activity (56), a phenomenon mediated by the inhibitory binding to phosphoinositides, especially phosphatidylinositol 4,5-bisphosphate (PIP2) [(57); Figure 1]. Intriguingly, membrane sequestered AMPD thereby forms a pool of inactive enzyme rapidly mobilizable by the action of phospholipases. In parallel to the well-known Gs-coupled β3-adrenoreceptor cascade, brown adipocytes strongly express Gq-coupled α1-adrenoreceptors that activate phospholipase C to hydrolyze PIP2 into inositol 1,4,5-trisphosphate and diacylglycerol (58). The second messenger inositol 1,4,5-trisphosphate is already responsible for the resulting increase in cytosolic calcium and thus for the sequestration of free UCP1-inhibitory nucleotides into non-inhibitory complexes (59). It is conceivable that in parallel, phospholipase C hydrolysis of PIP2 rapidly converts membrane-bound, inactive AMPD into soluble, active enzyme to decrease adenine nucleotide pool size in response to an adrenergic signal, as observed (23). If corroborated, pharmacological targeting of Gq-coupled α1-adrenoreceptors and their downstream signal transduction cascade may prove a valid alternative to the classical β-adrenergic pathway in the endeavor to identify safe activators of brown fat thermogenesis. For decades, it has been attempted to pharmacologically target the β3-receptor to treat aspects of human metabolic syndrome, to date without clinical breakthrough (60–62). The inherent challenge, i.e., unintended effects from receptor expression outside the target tissue, must be expected to be similar for α-agonists. Considered together with a completely different and partly unresolved intracellular second messenger cascade, α-adrenergic recruitment of nucleotide metabolism enzymes may prove a complementary approach worthwhile to follow.

## Summary and Outlook

The significance of purine nucleotides in the control of non-shivering thermogenesis was one of the first milestones in the mechanistic understanding of brown adipose tissue mitochondria, predating the identification of their target, uncoupling protein 1. As a means of acute thermogenic activation, nucleotides have long been neglected as passive inhibitors being displaced by actively regulated free fatty acid levels. In fact, cytosolic free purine nucleotide concentrations are adrenergically modified by several routes acting in concert, including calcium complex formation and enzymatic nucleotide degradation. The signaling cascade connecting norepinephrine and purine metabolic enzymes remains unresolved, and at least in the case of adenine nucleotides, plausibly relies on α1-adrenoreceptors and phospholipase C. Beyond the diligence work of elaborating these unknown pathways, the release of degradation products by active brown adipocytes presents the fascinating opportunity to discover novel markers and endogenous indicators of non-shivering thermogenesis (Figure 1). While the well-established endocrine/paracrine agents adenosine and ATP (63) are not detectably released from brown adipocytes, abundant guanosine, inosine, and AMP have all been reported to act as non-canonical transcellular messengers through known purinergic or novel receptors (64–69). Taken together, novel targets to manipulate or detect brown fat thermogenesis may be found both up- and downstream of the thermogenic modulation conferred by purine nucleotide metabolism.

## Author Contributions

AB-H and TF wrote this review article in concert. All authors read and approved the final manuscript.

### Conflict of Interest

The authors declare that the research was conducted in the absence of any commercial or financial relationships that could be construed as a potential conflict of interest.
